# BBCancer: an expression atlas of blood-based biomarkers in the early diagnosis of cancers

**DOI:** 10.1093/nar/gkz942

**Published:** 2019-10-29

**Authors:** Zhixiang Zuo, Huanjing Hu, Qingxian Xu, Xiaotong Luo, Di Peng, Kaiyu Zhu, Qi Zhao, Yubin Xie, Jian Ren

**Affiliations:** State Key Laboratory of Oncology in South China, Cancer Center, Collaborative Innovation Center for Cancer Medicine, School of Life Sciences, Sun Yat-sen University, Guangzhou 510060, China

## Abstract

The early detection of cancer holds the key to combat and control the increasing global burden of cancer morbidity and mortality. Blood-based screenings using circulating DNAs (ctDNAs), circulating RNA (ctRNAs), circulating tumor cells (CTCs) and extracellular vesicles (EVs) have shown promising prospects in the early detection of cancer. Recent high-throughput gene expression profiling of blood samples from cancer patients has provided a valuable resource for developing new biomarkers for the early detection of cancer. However, a well-organized online repository for these blood-based high-throughput gene expression data is still not available. Here, we present BBCancer (http://bbcancer.renlab.org/), a web-accessible and comprehensive open resource for providing the expression landscape of six types of RNAs, including messenger RNAs (mRNAs), long noncoding RNAs (lncRNAs), microRNAs (miRNAs), circular RNAs (circRNAs), tRNA-derived fragments (tRFRNAs) and Piwi-interacting RNAs (piRNAs) in blood samples, including plasma, CTCs and EVs, from cancer patients with various cancer types. Currently, BBCancer contains expression data of the six RNA types from 5040 normal and tumor blood samples across 15 cancer types. We believe this database will serve as a powerful platform for developing blood biomarkers.

## INTRODUCTION

The early detection of cancer can greatly reduce the probability of distance metastasis, thereby improving the survival rate of cancer patients (1). The measures of early detection include screening of cancer cells or tissues before symptoms are present and recognizing early symptoms before cancer progression. While a number of methods for early detection of cancer are proposed, only a handful of cancer screening methods are shown to be effective in the clinic. In recent years, liquid biopsy methods (mainly blood-based tests) for the early detection of cancer have received much attention (2). The data on blood-based tests are promising, but the specificity and sensitivity are still challenging (3,4). An ideal set of biomarker molecules and effective algorithms are needed to develop an accurate blood-based testing method.

Current blood-based tests mainly focused on molecules, such as circulating tumor DNAs (ctDNAs), with DNA fragments released by tumor cells or tissues into the blood circulating system, since these molecules could survive long in blood and contain genetic changes identical to the tumors they derive from. Genetic changes in ctDNAs, such as mutations, copy number changes, gene fusion events, and even DNA methylation patterns, have been widely investigated as biomarkers in blood-based tests. For instance, mutations in the ER gene can be frequently found in ctDNAs of breast cancer patients and can potentially be used as a biomarker to predict the response of endocrine therapy (5). Patients with innate trastuzumab resistance presented high HER2 copy number alterations in ctDNAs from HER2-positive gastric cancer patients (6). Xu et al constructed a prediction model based on ctDNA methylation for the diagnosis of hepatocellular carcinoma with high diagnostic specificity and sensitivity (7).

In contrast, RNA molecules have not received much attention as biomarkers for the early detection of cancers, since RNA molecules are unstable in the circulating system. However, an increasing number of studies have indicated that RNA molecules, such as microRNAs (miRNAs), circular RNAs (circRNAs), tRNA-derived fragments (tRFRNAs) and Piwi-interacting RNAs (piRNAs), are frequently detected in human blood (8–11), suggesting the promising prospect of these RNA molecules as biomarkers for blood-based tests in early cancer detection. Moreover, extracellular vesicles (EVs) and circulating tumor cells (CTCs) in the blood play fundamental roles in cancer progression. The coding and noncoding RNA molecules present in EVs and CTCs could potentially be used as biomarkers in blood-based tests.

Advances in high-throughput technologies, such as microarrays and next-generation RNA sequencing (RNA-Seq), have resulted in large amounts of gene expression data of blood samples across different cancer types. Some databases based on the integration of high-throughput data analysis have emerged in recent years. Kim *et al.* constructed EVpedia (12), a useful resource to elucidate the fundamental roles of EVs derived from prokaryotes and eukaryotes from 130 high-throughput sequencing studies. Li *et al.* developed exoRBase that contains 58 330 circRNAs, 15 501 lncRNAs and 18 333 mRNAs from 87 blood exosomal RNA-seq datasets (13). Nevertheless, a comprehensive online repository for these blood-based high-throughput gene expression data specialized for the early detection of cancer is still lacking.

**Figure 1.**
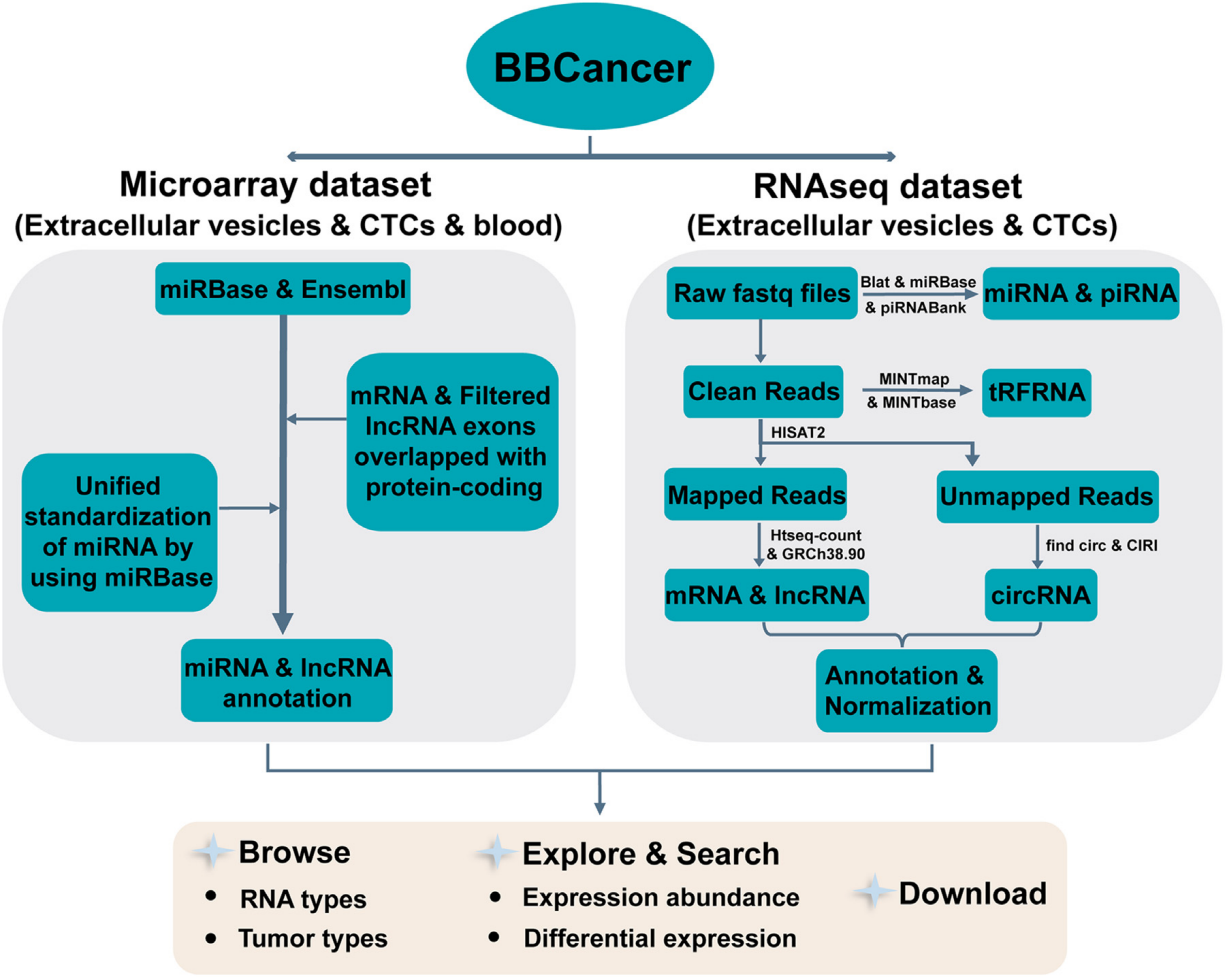
Overall design and construction of BBCancer.

Here, we present BBCancer (http://bbcancer.renlab.org/), a web-accessible and comprehensive open resource for providing the expression landscape of six RNA types, including mRNAs, lncRNAs, miRNAs, circRNAs, tRFRNAs and piRNAs in blood samples, including plasma, CTCs and EVs, from cancer patients with various cancer types (Figure 1). Using BBCancer, users are able to evaluate the expression abundance of RNAs of interest in tumor and normal blood samples from different cancer types. Moreover, users can explore the differential expression of RNAs of interest between tumor and normal blood samples. We believe that BBCancer will serve as a powerful platform for developing blood-based biomarkers for the early detection of cancers.

## MATERIALS AND METHODS

### Data sources

The RNA-seq and gene expression microarray raw datasets were collected from NCBI SRA (14) and GEO (15) databases. We queried the SRA and GEO databases using both cancer-related key words and blood-related key words. The SRA files from the SRA database were downloaded by the Aspera high-speed file transfer protocol (http://asperasoft.com/) and were converted to FASTQ files by using SRA Toolkit (SRA Toolkit Development Team, http://ncbi.github.io/sra-tools/). Sample information, such as tissue types (tumor or normal), was collected and manually curated. We also downloaded the probe sequences of each microarray platform from the GEO database or the websites of their corresponding companies for the purpose of reannotating the probes. All collected datasets were limited to human studies. Currently, all datasets were collected before July 2018.

### Analysis of RNA-seq data

All collected RNA-seq datasets were processed using the following pipeline. Briefly, FastQC was used to check the quality of raw sequencing data in the FASTQ format. The raw sequencing reads were trimmed by removing adapters and low-quality bases using Cutadapt (v. 1.7.1) (16). For long RNAs, the trimmed sequencing reads were mapped to the human reference genome (GRCh38) using HISAT2 (v. 2.0.4) (17). HTSeq-count (v. 0.6.0) (18) was employed to quantify the number of reads aligned to regions of mRNAs or lncRNAs. Read counts for each gene were normalized to the RPKM values (Reads Per Kilobase per Million mapped reads) (19). CircRNAs were identified using CIRI (v. 2.0.5) (20) and find_circ (v. 1.2) (21). Only circRNAs that were identified by either of the two tools were retained. Read counts for each circRNA were normalized by calculating the RPM values (Read counts per million mapped reads). For miRNAs and piRNAs, all of the small sequencing reads were aligned to the miRBase (v. 22) and piRNABank (22) using a BLAST-like alignment tool (BLAT) (23). Only alignments with no more than one mismatch, no gaps and a mapping length equal to the length of the small RNA were considered as the best match. The raw counts of miRNAs and piRNAs were normalized to RPM values (24). For tRFRNAs, all of the small sequencing reads were analyzed using MINTmap (v. 1.0) (25) and MINTbase (26) to identify and quantify tRFRNAs.

### Analysis of gene expression microarray data

Previous studies have shown that the probes of many protein-coding gene expression microarrays should be reannotated to lncRNAs according to the latest gene annotation files. We used the following strategy to reannotate the probes from different gene expression microarray platforms. The latest gene annotation files were downloaded from the ENSEMBL database. A BLAST-like alignment tool (BLAT) (27) was used to align all probe sequences from the different microarray platforms to the human genome (GRCh38). Only alignments with no more than one mismatch, no gaps and a similarity score larger than 90 bp were retained. In general, probes (50–60 nucleotides) from Agilent and Illumina platforms were designed to locate target genes or transcripts. The microarrays from Affymetrix utilized a probe set containing a group of 25-mer probes to represent a gene or transcript. Thus, for Affymetrix data, we combined probes that specifically corresponded to the same probe set and ensured that there were at least three perfectly matching and adjacent probes in each probe set. Second, we mapped the probes to coding genes or lncRNAs according to their genomic coordinates. If any probes were targeted to both coding genes and lncRNAs, then we only preserved the annotation of the coding genes. MicroRNA probes were uniformly annotated by miRBase (v22).

The following strategy of gene expression was applied to the diverse microarray platforms within BBCancer. First, the raw data were normalized using different methods according to the different platforms. For Agilent, Illumina and other platforms, the ‘Limma’ package (28) was employed to quantile-normalized the data sets. For the Affymetrix GeneChip data, raw CEL files were normalized with the RMA algorithm (‘affy’ package v. 1.26 or ‘apt-probeset-summarize’ in Affymetrix Power Tools v. 1.19.0) (29,30). For certain studies lacking raw data, we used the data matrix provided in the GEO database as a normalized expression matrix. The expression of each gene was log_2_ transformed. Next, all of the probes were transformed to gene names based on the above re-annotation file. The average expression was calculated to represent those genes that are targeted by multiple probes. Finally, a robust rank aggregation algorithm was utilized to integrate the rank of gene expression abundance from multiple datasets for the same cancer type.

### Differential expression analysis

We categorized differential expression with one of the following conditions: tumor versus normal, precancerous lesion versus normal and precancerous lesion versus tumor. For RNA-Seq and microarray data, differential expression analysis was performed with the ‘DESeq2’ (31) and ‘Limma’ package (32), respectively. Finally, a robust rank aggregation algorithm was utilized to integrate the blood-basedRNA profiles in a unbiased manner (33). The aggregation rank score (AR score) represents the integrated rank from the meta-analysis of the fold-changes in different studies. All results were scaled by cancer type and presented in a heat map, allowing users to interactively explore the expression of RNAs of interest.

### Database and web interface implementation

All data in BBCancer were stored and managed by MySQL tables. The web interfaces were implemented in Hyper Text Markup Language (HTML), Cascading Style Sheets (CSS) and JavaScript (JS). To visualize the analysis results, multiple statistical diagrams were embedded in the website. The interactive heat maps showing the expression abundance and differential expression were constructed by DataTables. The charts presenting gene expression rank and the boxplots showing the differential expression were drawn by Highcharts. Furthermore, all analyses in the BBCancer website were performed in R.

## RESULTS

### Database content

In the current release, BBCancer contains expression data for RNA molecules from 7,184 samples, including 5,040 blood samples such as extracellular vesicles (EVs) and circulating tumor cells (CTCs), in normal person and 15 cancer types including breast cancer, borderline ovarian tumor, cervical cancer, colorectal cancer, esophageal cancer, gastric cancer, liver cancer, lung cancer, pancreatic cancer, and prostate cancer (Table 1). Six types of RNA (19 612 mRNAs, 10 918 lncRNAs, 60 306 circRNAs, 2568 miRNAs, 1231 piRNAs and 43 459 tRFRNAs) were included in BBCancer (Table 2). To our knowledge, BBCancer represents the largest blood sample resource for cancer biomarker research.

**Table 1.** Summary of the gene expression data of samples collected in BBCancer

	Extracellular vesicles	Circulating tumor cells	Other blood	Tumor tissue	Normal tissue	Precancerous tissue
**Borderline ovarian tumor**	0	0	66	0	0	0
**Breast cancer**	42	112	171	463	149	0
**Colorectal cancer**	215	6	149	113	271	41
**Esophageal cance**r	28	0	88	117	118	6
**Gastric cancer**	0	0	115	22	31	8
**Glioblastoma**	41	0	0	18	69	0
**Liver cancer**	21	0	81	68	77	5
**Lung cancer**	3	0	123	47	39	0
**Multiple myeloma**	10	0	9	53	67	0
**Ovarian cancer**	0	0	320	8	4	0
**Pancreatic cancer**	36	24	115	20	5	0
**Sarcoma**	0	0	115	19	4	0
**Wilms' tumor**	0	0	15	60	4	0
**Prostate cancer**	59	0	0	29	128	0
**Renal cell carcinoma**	41	0	0	18	63	0
**Normal person**	167	25	2843	NA	0	0

**Table 2.** Summary of the gene number for different RNA types in blood samples in BBCancer

	mRNA	lncRNA	circRNA	miRNA	piRNA	tRFRNA
**Breast cancer**	17 313	6036	0	2467	765	1905
**Colorectal cancer**	19 307	5791	40 564	2483	374	32 623
**Glioblastoma**	16 557	6818	0	0	0	0
**Lung cancer**	17 237	528	0	2310	0	0
**Liver cancer**	16 903	4894	54 481	1630	0	0
**Prostate cancer**	0	0	0	971	176	23 580
**Pancreatic cancer**	17 286	6417	41 105	1958	114	9289
**Multiple myeloma**	0	0	0	1302	528	10 838
**Renal cell carcinoma**	0	0	0	1732	214	13 953
**Sarcoma**	0	0	0	1634	0	0
**Wilms' tumor**	0	0	0	1198	0	0
**Borderline ovarian tumor**	0	0	0	1634	0	0
**Esophageal cancer**	0	0	0	1634	0	0
**Gastric cancer**	0	0	0	1634	0	0
**Ovarian cancer**	0	0	0	1634	0	0
**Normal person**	19 245	3385	39 358	2568	400	28 581

### Web interface and usage

BBCancer provides a user-friendly web interface that contains four modules: Explore, Browse, Statistics and Download.

#### Explore

**Figure 2.**
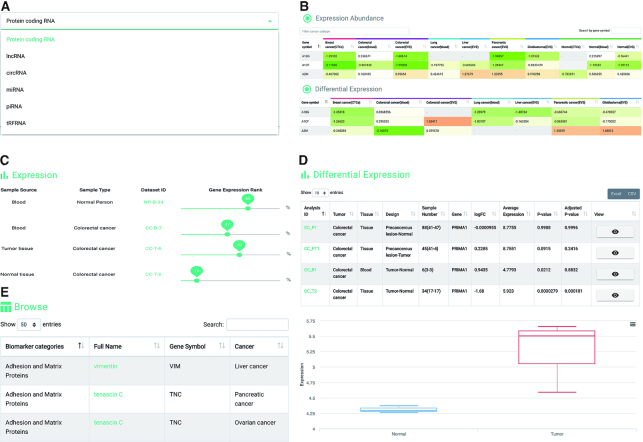
A schematic workflow of the search interface in BBCancer. (**A**) BBCancer contains six types of RNAs (mRNA, lncRNA, circRNA, miRNA, piRNA and tRFRNA). (**B**) An interactive heat map showing the meta score of the gene expression fold change for each RNA type between tumor blood samples and normal blood samples from different studies for each cancer type (upper panel), and an interactive heat map showing the meta score of the gene expression rank for each RNA type in tumor and normal blood sample for each cancer type **(**lower panel**)**. (**C**) Snapshot of the search results for ‘*TIMP1*’ using the ‘Protein coding’ search mode. Detailed information on the expression abundance of *TIMP1* in all the colorectal cancer datasets is shown in the gene expression rank line graphs. (**D**) The expression of RNA in different studies of colorectal cancer compared to normal tissues. Click the ‘view’ button to obtain the boxplot representing the differential expression of RNAs between different conditions. (**E**) Interactive table for browsing the known blood biomarkers collected from Uttle et al (34).

In BBCancer, six RNA types, including mRNA, lncRNA, circRNA, piRNA, miRNA and tRFRNA, were provided (Figure 2A). An ideal blood biomarker requires two characteristics: distinguishable and detectable. First, the presence of biomarkers in cancer patients should be distinguished from that in normal persons. In BBCancer, we implemented a module, named ‘Differential expression’, to help users find biomarker candidates with such characteristics. The ‘Differential expression’ module allows users to explore the differential expression of RNA of interest between tumor and normal blood samples by an interactive meta-score heat map (Figure 2B). Another desired characteristic of an ideal biomarker is detectable. For this end, we implemented a module, named ‘Expression abundance’, to help users evaluate the expression abundance of an RNA of interest. In the ‘Gene expression abundance’ module, an interactive heat map was implemented to present the meta-score from differential studies for the same cancer type (Figure 2B). In the interactive heat maps of the two modules, a selection box was provided to help users quickly locate cancers, and a text box was provided to allow users to search RNAs of interest (Figure 2B). Furthermore, users can sort the RNAs by clicking the column name. The detailed information for an RNA of interest in a certain cancer was shown when clicking the ‘meta-score’ box. In the detailed information page, gene expression rank line graphs were provided to show the expression abundance of the selected RNAs in all datasets categorized as normal blood samples, tumor blood samples, normal tissues, tumor tissues and precancerous tissues (Figure 2C). In addition, a table embedded with boxplots was provided to show the differential expression of the selected RNAs in different studies, including comparisons between tumor blood samples and normal blood samples, tumor tissue samples and normal tissue samples, and precancerous tissue samples and normal tissue samples (Figure 2D).

#### Browse

BBCancer systematically categorized the known blood biomarker by ‘Biomarker categories’, ‘Biomarker’, ‘Gene Description’ and ‘Cancer Type’ (Figure 2E). Users are allowed to explore the blood molecular marker of interest to obtain the results of gene expression abundance and differential expression analysis.

#### Statistics

Detailed statistics on the data in BBCancer were provided in the ‘Statistics’ module.

#### Download and Help

All data in the database can be downloaded from the ‘Download’ page, and a detailed introduction to the BBCancer database as well as a tutorial are available on the ‘Help’ page.

### Systematical screening of potential blood biomarkers using BBCancer

**Figure 3.**
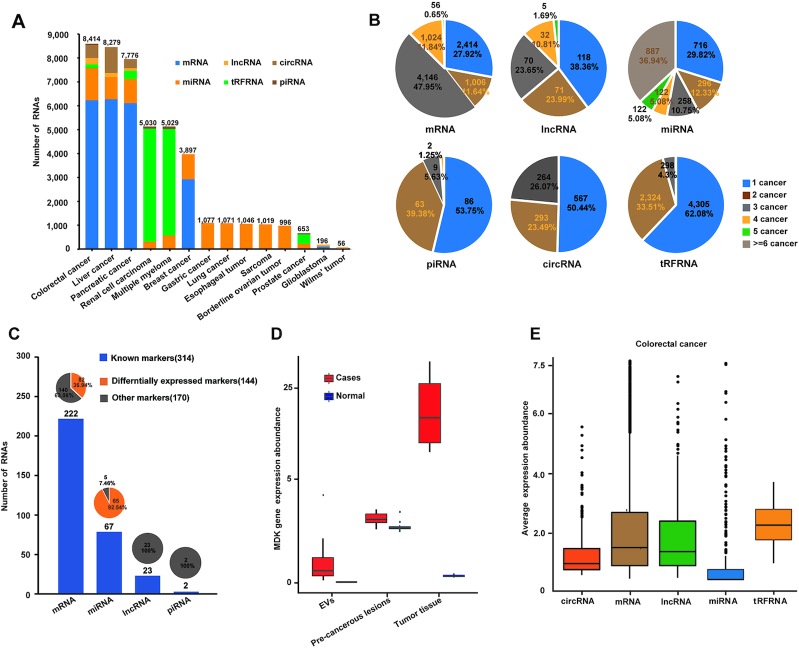
Systematical screening of potential blood biomarkers. (**A**) A bar chart showing the differentially expressed RNAs between tumor blood samples and normal blood samples in various cancer types. (**B**) A pie chart showing the number of differentially expressed genes shared by different cancers. (**C**) A bar chart showing the known biomarkers with higher expression in tumor blood samples compared to normal blood samples. (**D**) *MDK* expression was higher in blood samples of liver cancer patients compared to blood samples of normal persons (fold change >1.5, adjusted *P*-value < 0.05). (**E**) A box plot showing the expression abundances of five different RNA types in colorectal cancer blood samples.

We next systematically evaluated the utility of BBCancer in discovering potential blood biomarkers for the early detection of cancer. To this end, we first explored the differentially expressed RNAs between tumor and normal blood samples across various cancer types, which resulted in 8646 mRNAs, 296 lncRNAs, 1124 circRNAs, 2401 miRNAs, 160 piRNAs and 6935 tRFRNAs (Figure 3A, fold change > 1.5, adjusted *P*-value < 0.05). A number of RNAs were differentially expressed in more than five types of cancers, suggesting broad pancancer functional importance of these RNAs (Figure 3B). Uttle *et al.* identified 788 potential blood biomarkers covering 13 cancer types based on a comprehensive investigation of 3990 related papers (34). We examined the expression pattern of these 788 potential biomarkers across different cancer types in BBCancer. Among the 788 biomarkers, 314 have expression data in BBCancer. We found that 144 (82 mRNAs, 62 miRNAs) out of 314 biomarkers had significantly higher expression in tumor blood samples compared to normal blood samples in at least one cancer type (Figure 3C). For example, *MDK* was recently reported to be a promising blood biomarker for the diagnosis of liver cancer (35). In BBCancer, we found *MDK* expression was higher in blood samples of liver cancer patients compared to blood samples of normal persons (fold change >1.5, adjusted *P*-value < 0.05) (Figure 3D). Moreover, *MDK* expression was also significantly higher in precancerous lesions compared to normal tissues (Figure 3D, fold change >1.5, adjusted *P*-value < 0.05). These results suggest that *MDK* is an ideal biomarker for the early detection of liver cancer.

**Figure 4.**
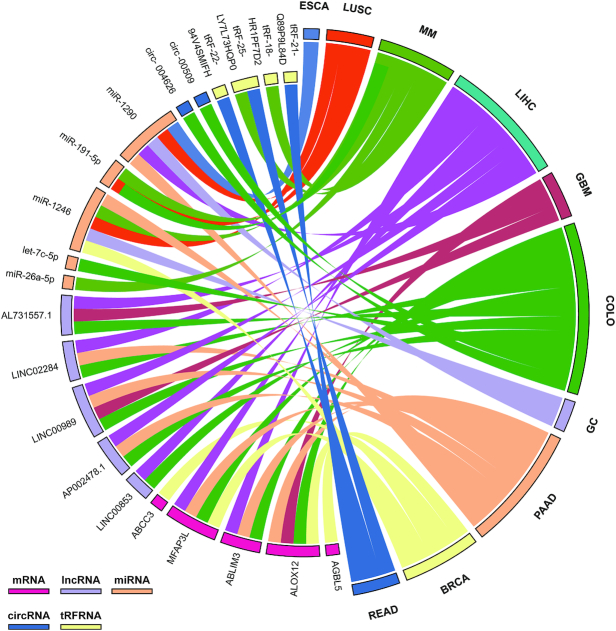
Top 5 circulating RNAs of each RNA type that are significantly overexpressed in the blood samples of different cancers compared to normal blood samples.

By comparing the expression abundances of six different RNA types in blood samples, we found that the expression abundances of tRFRNAs were highest among the five RNA types in colorectal cancer, suggesting the promising prospects of tRFRNAs as a blood biomarker for the early detection of colorectal cancers (Figure 3E). To provide a list of promising blood biomarkers for each cancer type, we further filtered the differentially expressed RNAs using expression abundance. The top 5 differentially expressed RNAs for each RNA type with the highest expression abundance in each cancer are shown (Figure 4). For instance, the expression of *ABCC3* in patients with nonsmall cell lung cancer was potentially correlated with drug resistance (36). The high expression level of exosome *mir-1246* in breast cancer has been reported to be related to breast tumor progression (37). Furthermore, studies on bladder cancer have shown a negative correlation between the high expression of *mir-26a-5p* and patient survival (38). These results suggest that these circulating RNAs may be potential blood biomarkers for related cancers. Overall, the BBCancer database contains a number of clinically relevant potential circulating RNAs that can assist researchers in screening blood molecular markers.

## DISCUSSION

BBCancer is a comprehensive open resource for providing the expression landscape of RNA molecules derived from blood samples, including plasma, CTCs and EVs, from cancer patients with various cancer types.

Compared to other existing circulating RNA resources, such as exoRbase and EVpedia, BBCancer is the first database focusing on the expression atlas of RNA molecules in blood samples of cancer patients. Briefly, BBCancer has the following advantages: (i) BBCancer contains many types of RNA. Currently, six types of RNA (mRNA, lncRNA, miRNA, circRNA, tRFRNA and piRNA) were included. (ii) BBCancer holds the largest number of blood samples, with 5,040 normal and tumor blood samples across 15 cancer types. (iii) BBCancer allows users to rapidly and interactively explore the expression abundance or differentially expressed RNAs in different studies by meta-analysis, promoting the discovery of RNA biomarkers in cancers of interest.

In conclusion, we believe that BBCancer will be of significant benefit to the community and boost further advances in developing blood-based biomarkers for the early detection of cancer.
